# Spondylodiskitis and endocarditis due to *Streptococcus gordonii*

**DOI:** 10.1186/s12941-017-0243-8

**Published:** 2017-10-04

**Authors:** Ziv Dadon, Assaf Cohen, Yael M. Szterenlicht, Marc V. Assous, Yair Barzilay, David Raveh-Brawer, Amos M. Yinnon, Gabriel Munter

**Affiliations:** 1 Department of Internal Medicine A, Shaare Zedek Medical Medicine, Affiliated with the Hebrew University-Hadassah Medical School, Jerusalem, Israel; 2 Department of Internal Medicine C, Shaare Zedek Medical Medicine, Affiliated with the Hebrew University-Hadassah Medical School, Jerusalem, Israel; 3 Clinical Microbiology and Immunology Laboratory, Shaare Zedek Medical Medicine, Affiliated with the Hebrew University-Hadassah Medical School, Jerusalem, Israel; 4 Spine Unit of the Department of Orthopedics, Shaare Zedek Medical Medicine, Affiliated with the Hebrew University-Hadassah Medical School, Jerusalem, Israel; 5 Infectious Disease Unit, Shaare Zedek Medical Medicine, Affiliated with the Hebrew University-Hadassah Medical School, Jerusalem, Israel; 6 Division of Internal Medicine, Shaare Zedek Medical Medicine, Affiliated with the Hebrew University-Hadassah Medical School, P.O. Box 3235, 91031 Jerusalem, Israel

**Keywords:** Spondylodiskitis, Endocarditis, *Streptococcus gordonii*, Duration of antibiotic treatment

## Abstract

**Background:**

*Streptococcus gordonii* is an infrequent cause of infective endocarditis (IE); associated spondylodiskitis has not yet been described in the literature.

**Purpose:**

We describe 2 patients who presented with new-onset, severe back pain; blood cultures revealed *S. gordonii* bacteremia, which led to the diagnosis of spondylodiskitis and IE. We review our 2-decade experience with *S. gordonii* bacteremia to describe the clinical and epidemiological characteristics of these patients.

**Results:**

In our hospital over the last 20 years (1998–2017), a total of 15 patients with *S. gordonii* bacteremia were diagnosed, including 11 men and 4 women, and the mean age was 65 ± 22 (range 23–95). The most common diagnosis was IE (9 patients), spondylodiskitis (the presented 2 patients, who in addition were diagnosed with endocarditis), necrotizing fasciitis (1), sternitis (1), septic arthritis (1) and pneumonia (1). The 11 patients with IE were treated with penicillin ± gentamicin, or ceftriaxone for 6 weeks, 5 required valve surgery and 10/11 (91%) attained complete cure. The 2 patients with diskitis required 2–3 months of intravenous antibiotics to achieve complete cure.

**Conclusion:**

Spondylodiskitis was the presenting symptom of 2/11 (18%) patients with *S. gordonii* endocarditis. Spondylodiskitis should probably be looked for in patients diagnosed with *S. gordonii* endocarditis and back pain as duration of antibiotic treatment to achieve complete cure may be considerably longer.

## Background

The Viridians group streptococci are a group of Gram-positive cocci, composed of heterogeneous groups of organisms with complex taxonomy [1]. They are sub-classified into six major groups, including the *S*. *mutans* group, *S*. *mitis* group, *S*. *anginosus* group, *S*. *salivarius* group, *S*. *bovis* group and *S*. *sanguinis* group [2, 3]. The *S*. *sanguinis* group includes *S*. *sanguinis*, *S*. *parasanguinis*, and *Streptococcus gordonii* [3]. The Viridans streptococci are pathogens with low virulence that are generally present in the mouth and upper airways, the gastrointestinal and the female genital tract. These organisms infrequently cause invasive infections such as orbital cellulitis and endophthalmitis, pneumonia and bacteremia, endocarditis, meningitis and toxic shock-like syndrome [1]. The clinical presentation of *S*. *gordonii* infection may present as infective endocarditis (IE) [4, 5], septic arthritis [6, 7] and spontaneous bacterial peritonitis [8, 9].

During the last 2 years we took care of two non-related, elderly male patients, who presented with severe back pain of recent onset. Spondylodiskitis was diagnosed by CT and/or MRI, blood cultures were obtained (although only one patient had fever) which revealed *S. gordonii* and intravenous antibiotic treatment was started. There were no additional stigmata of endocarditis, but because of this organism’s tendency to cause endocarditis, trans-esophageal echocardiography was obtained, which revealed vegetations in both patients. Prolonged and severe back pain in both patients led to intense discussion regarding the duration of antibiotic treatment. Recent guidelines suggest that for most patients with diskitis, mostly due to *Staphylococcus aureus*, a 6 weeks’ course suffices and leads to complete cure. An extensive literature search did not reveal any reports on *S. gordonii* spondylodiskitis with information on the course of this infection and possible need for longer treatment. Therefore, we reviewed our own database to describe the epidemiology and clinical characteristics of patients with *S. gordoni* bacteremia, focusing on endocarditis and spondylodiskitis.

## Patient 1

A 63 year-old male was admitted because of increasing low back pain of 2 weeks’ duration, which worsened while lying supine and changing position. In addition, he reported a low grade fever of 38 °C. The patient suffered from poor dental hygiene and 3 weeks earlier he underwent periodontal debridement. His past medical history was significant only for bipolar disease treated with lithium.

The physical examination revealed a 38 °C temperature, a 2/6 systolic murmur with maximal intensity at the apex and significant lower back midline tenderness. There were no stigmata of endocarditis, such as Osler’s nodes, Roth’s spots or splenomegaly. Laboratory testing revealed normocytic anemia (Hb 11.2 g/dl), a peripheral white cell count of 16,600/µl (with 82% neutrophils), an erythrocyte sedimentation rate (ESR) of 89 mm, C-reactive protein (CRP) 13 mg/dl (normal up to 5), AST 54 IU/l and ALT 73 IU/l. A CT scan demonstrated L4–5 frontal disc herniation. An urgent lumbar spine MRI ruled out abscess, but showed wide and thick enhancement of the epidural and paravertebral tissues, mainly on the left side, suggesting soft tissue infection, with local intervertebral space disc edema. One day after admission, two blood cultures yielded alpha-haemolytic streptococci, subsequently identified by MALDI-TOF (matrix assisted laser desorption/ionization time of flight mass spectrometry) as *S. gordonii*, with a minimal inhibitory concentration (MIC) of 0.06 µg/ml penicillin. Transesophageal echocardiography (TEE) demonstrated a mobile vegetation of 13 mm attached to the aortic valve without valvular dysfunction.

With a diagnosis of aortic endocarditis with spondylodiskitis due to *S. gordonii* the patient was started on continuous, intravenous (IV) penicillin 18 million units/day for at least 6 weeks and gentamicin 3 mg/kg/days for the first 2 weeks. Subsequent blood cultures remained negative, but back pain progressed with new onset severe constipation. Repeat MRI showed worsening of the local findings. After several weeks of in-hospital treatment, with a gradual decrease in CRP and ESR and shrinkage (to 6 mm) of the vegetation on a repeat TEE, the patient was discharged for home IV treatment. Throughout this period he continued to complain of low back pain with onset of radiation to his left leg, for which he was readmitted after 2 weeks. Neurologic examination was normal. A repeat MRI showed expansion of prior findings, L4–5 vertebral lysis and necrotic intervertebral changes. Throughout the patient’s admission and subsequent follow-up, repeat interdisciplinary discussions were conducted to consider surgical debridement and stabilization of the involved vertebrae. At this stage an intervertebral biopsy was performed, but remained sterile. Intravenous penicillin was continued for a total of 6 weeks, with additional 4 weeks of oral treatment. CRP and ESR gradually decreased and have remained within normal limits after 4 months of follow-up. Back pain has slowly improved throughout this period.

## Patient 2

An 82 year-old male was admitted due to low back pain of 2 weeks’ duration. The pain radiated to his left leg, causing muscular weakness. There was no history of fever or trauma. Five years earlier he underwent a biological aortic valve replacement and repair of his mitral valve. In addition, he suffered from Parkinson’s disease, hypothyroidism and hyperparathyroidism.

The physical examination revealed a 36.7 °C temperature, severe lumbar back pain, and weakness of the iliopsoas and quadriceps muscles. Laboratory testing showed normocytic anemia (Hgb 13 g/dl), a peripheral white cell count of 11,800/µl (with 85% neutrophils), an ESR of 50 mm, CRP 13.5 mg/dl, AST 52 IU/l and ALT 75 IU/l. A CT scan demonstrated severe degenerative changes, a herniated L4–5 disc, a calcified L5–S1 disc and scoliosis without evidence of bone destruction. The MRI showed an epidural abscess at L5–S1 level compressing the L5 nerve root on the left, para-vertebral enhancement and abscess at the left lateral recess, posterior to S1. Three blood cultures yielded *S. gordonii* identified by MALDI-TOF. The patient was started on continuous, intravenous penicillin 24 million units/day. Because of the patient’s biological valve, a TEE was performed, which demonstrated a 14 mm vegetation attached to the posterior leaflet of the mitral valve. The patient was discharged to complete home treatment with intravenous penicillin for 8 weeks.

After 2 months, inflammatory markers remained high (CRP 30 mg/dl, ESR 60 mm). An MRI was performed showing the disappearance of the epidural lesion, but evidence of L5 osteomyelitis. Repeat blood cultures were negative. A CT-guided biopsy was performed of the L5–S1 disk: no histologic inflammation was detected, and the bone and disk cultures were negative. At 3-month follow-up, the patient reported a significant improvement in back pain. After 6 months the CRP and ESR had completely normalized.

## Discussion

Although recent guidelines question the need for antibiotic treatment longer than 4–6 weeks for infectious diskitis, our experience with these 2 patients with *S. gordonii* endocarditis and associated diskitis suggests otherwise [10]. In both instances antibiotic treatment was discontinued after 6 weeks, which is definitely sufficient treatment for streptococcal endocarditis. However, in both cases back pain waxed and waned, never disappeared, and after short intervals increased with vengeance. Both patients described their pain as excruciating with an intensity of 7–9 out of 10. Repeat MRI revealed worsening of signs, and the combination of continued, severe pain, persisting high inflammatory markers (such as CRP and ESR) and worsening MRI findings led to a second 6-week course of intravenous antibiotics. In both cases, the second course was accompanied by a gradual decrease and normalization of inflammatory markers and improved MRI findings. Back pain improved gradually over time, but in both patients continued for several months after discontinuation of antibiotic treatment—and was probably related to the destruction of the motion segment.

In our hospital over the last 20 years (1998–2017), a total of 15 patients with *S. gordonii* bacteremia were diagnosed, including 11 men and 4 women, and the mean age was 65 ± 22 (range 23–95) (Table 1). The most common diagnosis was IE (9 patients), spondylodiskitis (the presented 2 patients, who in addition were diagnosed with endocarditis), necrotizing fasciitis (1), sternitis (1), septic arthritis (1) and pneumonia (1). The 11 patients with IE were treated with penicillin for 6 weeks with gentamicin for 2 weeks, or ceftriaxone for 6 weeks. Five of the IE patients underwent valve surgery and 10/11 (91%) attained complete cure. As mentioned, the two patients with spondylodiskitis required 2–3 months of intravenous antibiotics to achieve complete cure of their spinal infection.

**Table 1:** All patients with *Streptococcus gordonii* bacteremia in one medical center, 1998–2017

No.	Year	Gender/age	Presenting symptoms	Diagnosis	Diagnostic modality	Antibiotic treatment + duration	Surgery?	Outcome
1	1998	F/23	Fever	IE (MV)	TEE	IV gentamicin (2 week) IV penicillin (4 week)	No	Cure
2	2005	M/95	Septic arthritis left shoulder	Septic arthritis	TTE, CT	Unknown	No	Died
3	2006	M/37	Exertional dyspnea	IE (AV)	TTE	Unknown	Yes, AVR	Died
4	2006	F/50	Fever	Necrotizing fasciitis	TEE, CT	IV penicillin + gentamicin; IV tazocin + clindamycin	Yes	Complicated course. Died after 6 months
5	2006	M/45	Fever	IE (MV)	TEE	IV penicillin (6 week) + gentamicin (1 week)	Yes, MVR	Cure
6	2007	M/75	Fever	IE (MV + AV)	TEE	IV penicillin (6 wk)	No	Cure
7	2010	M/79	Fever following AVR and MVR	Sternitis	TEE	IV ceftriaxone (4 weeks)	No	Cure
8	2013	F/83	Fever	IE (MV)	TEE + CT	IV ceftriaxone (6 week)	No	Cure
9	2014	M/78	FUO	IE (MV + AV)	TEE + CT	IV penicillin (6 week)	Yes, AVR + MVR	Cure
10	2014	M/71	Fever	IE (clinical)	TEE	IV penicillin (6 week)	No	Cure
11	2015	M/31	Fever	IE (MV)	TTE	IV penicillin (4 week)	Yes, MVR	Cure
12	2016	M/82	Back pain	Diskitis + IE (MV)	TEE + MRI + CT	Two courses of IV penicillin (each 6 week)	No	Cure
13	2016	F/89	Fever	Pneumonia	TEE	IV ceftriaxone (2 week)	No	Cure
14	2016	M/63	Back pain	Diskitis + IE (AV)	TEE + MRI + CT	IV penicillin (8 week) + oral amoxycyline (4 week)	No	Cure
15	2017	M/71	General deterioration	IE (AV)	TEE	IV penicillin (6 week)	Yes, AVR + MVR	Cure

AV, aortic valve; CT, computerized tomography; F, female; FUO, fever of unknown origin; IE, infective endocarditis; IV, intravenous; M, male; MRI, magnetic resonance imaging; MV, mitral valve; TEE, trans-esophageal echocardiogram; TTE, trans-thoracic echocardiogram

We recently described 2 patients with enterococcal diskitis and associated endocarditis, and reviewed 20 similar patients reported as case reports in the literature [11]. Although some of these patients received 2–3 months of intravenous antibiotic treatment, most only received the usual 6 weeks course indicated for enterococcal endocarditis—their course was significantly more benign, with much less intensive back pain, less motion segment destruction, and faster resolution of the inflammatory markers. Recently, an animal model of endocarditis with *S. gordonii* revealed that no fewer than 13 bacterial genes were induced during endocarditis, probably contributing to increased virulence [12].

In conclusion, in this single-center experience with 15 patients suffering from *S. gordonii* bacteremia, spondylodiskitis was the presenting symptom of 2/11 (18%) patients with *S. gordonii* endocarditis. spondylodiskitis should probably be looked for in patients diagnosed with *S. gordonii* endocarditis suffering from back pain as duration of antibiotic treatment to achieve complete cure may be considerably longer than in the absence of spinal infection.
